# T1 mapping combined with arterial spin labeling MRI to identify renal injury in patients with liver cirrhosis

**DOI:** 10.3389/fendo.2024.1363797

**Published:** 2024-08-09

**Authors:** Shuangshuang Xie, Mengyao Chen, Chiyi Chen, Yumeng Zhao, Jiaming Qin, Caixin Qiu, Jinxia Zhu, Marcel Dominik Nickel, Bernd Kuehn, Wen Shen

**Affiliations:** ^1^ Radiology Department, Tianjin First Central Hospital, Tianjin Institute of Imaging Medicine, School of Medicine, Nankai University, Tianjin, China; ^2^ Liver Surgery Department, Tianjin First Central Hospital, School of Medicine, Nankai University, Tianjin, China; ^3^ MR Research Collaboration, Siemens Healthineers, Beijing, China; ^4^ MR Application Predevelopment, Siemens Healthcare GmbH, Erlangen, Germany

**Keywords:** cirrhosis, renal function, magnetic resonance imaging, arterial spin labeling, T1 mapping

## Abstract

**Purpose:**

We investigated the capability and imaging criteria of T1 mapping and arterial spin labeling (ASL) MRI to identify renal injury in patients with liver cirrhosis.

**Methods:**

We recruited 27 patients with cirrhosis and normal renal function (cirrhosis-NR), 10 with cirrhosis and renal dysfunction (cirrhosis-RD) and 23 normal controls (NCs). All participants were examined via renal T1 mapping and ASL imaging. Renal blood flow (RBF) derived from ASL was measured from the renal cortex, and T1 values were measured from the renal parenchyma (cortex and medulla). MRI parameters were compared between groups. Diagnostic performances for detecting renal impairment were statistically analyzed.

**Results:**

Cortical T1 (cT1) and medullary T1 (mT1) were significantly lower in the NCs than in the cirrhosis-NR group. The cortical RBF showed no significant changes between the NCs and cirrhosis-NR group but was markedly decreased in the cirrhosis-RD group. The areas under the curve (AUCs) for discriminating cirrhosis-NR from NCs were 0.883 and 0.826 by cT1 and mT1, respectively. Cortical RBF identified cirrhosis-RD with AUC of 0.978, and correlated with serum creatinine (r = −0.334) and the estimated glomerular filtration rate (r = 0.483). A classification and regression tree based on cortical RBF and cT1 achieved 85% accuracy in detecting renal impairment in the cirrhosis.

**Conclusion:**

Renal T1 values might be sensitive predictors of early renal impairment in patients with cirrhosis-NR. RBF enabled quantifying renal perfusion impairment in patients with cirrhosis-RD. The diagnostic algorithm based on cortical RBF and T1 values allowed detecting renal injury during cirrhosis.

## Introduction

Renal dysfunction (RD) is a common and critical complication in patients with liver cirrhosis (1). Acute kidney injury (AKI) occurs in approximately 47% of hospitalized patients with cirrhosis and 30% of outpatients with cirrhosis (2, 3). AKI incidence in patients with cirrhosis increases morbidity and mortality. Once AKI has been diagnosed, patients should immediately begin a specific pharmacological treatment and stop taking any drugs associated with AKI (4). Appropriate treatment can significantly improve renal function and short-term survival (5–7). Hence, early detection of renal injury will significantly benefit patients in their treatment and prognosis.

In clinical practice, sudden increases in serum creatinine (Scr) are the earliest clinical indicators for diagnosing AKI in cirrhosis; however, many factors influence Scr levels. Patients with cirrhosis exhibit impaired hepatic production of creatine, and Scr may lead to underestimating the severity of the reduced renal function. Additionally, reduced muscle mass and renal tubular secretion of creatinine in cirrhosis result in lower Scr levels, and hyperbilirubinemia due to liver injury in cirrhosis results in inaccurate measurements of Scr via calorimetric methods (8). Thus, a reliable, noninvasive method is needed to detect renal injury before abnormal Scr is detected.

The underlying pathophysiology of renal complications in cirrhosis is complex. A decreased effective arterial blood volume (EABV) due to splanchnic vasodilatation is the most common cause of these renal complications and can lead to renal vasoconstriction, followed by reduced renal blood flow (RBF) and impaired function (9). Arterial spin labeling (ASL) magnetic resonance imaging (MRI) allows measuring RBF without administering exogenous contrast agents (10). Previous studies with patients and animals have illustrated the ability of ASL MRI in detecting renal perfusion changes resulting from various etiologies (11–13). Additionally, various renal parenchymal injuries are frequently combined in patients with cirrhosis, such as acute tubular necrosis, tubular atrophy, interstitial inflammation and fibrosis (14). T1 mapping is a novel MRI technique that enables characterizing tissue with great specificity. Many factors influence T1 time, which depends on the molecular environment and number of water molecules within the tissue. Previous studies have demonstrated that T1 time can help detect renal injury (15–17).

To our knowledge, few reports have focused on using MRI to evaluate renal injury in patients with cirrhosis. Yu et al. (13) evaluated dynamic renal perfusion changes using ASL MRI and demonstrated significantly decreased RBF in rats with cirrhosis induced by common bile duct ligation. Qin et al (18). also demonstrated the decrease of RBF and increase of T2^*^ values of renal parenchyma in rats with cirrhosis induced by carbon tetrachloride. Özkök et al. (19) confirmed the value of renal T1 mapping in showing the early stages of renal parenchymal disease without apparent RD in patients with chronic hepatitis B viral infection. So, we hypothesis ASL combined with T1 mapping can used to detect hemodynamic and structural injuries of kidney in patients with liver cirrhosis. Here, we compare renal RBF and T1 values in healthy individuals and cirrhosis with or without RD. The purpose of this study was to investigate the feasibility of ASL and T1-mapping MRI to detect early renal impairment and value of these techniques in diagnosing RD in patients with cirrhosis.

## Materials and methods

### Participants

The local ethics committee approved this prospective study, and all patients provided informed consent. Between March 2019 and July 2022, 43 patients who received a definitive clinical diagnosis of chronic liver disease and an imaging diagnosis of liver cirrhosis at our hospital were enrolled. Alanine transaminase (ALT), aspartate aminotransferase (AST), total bilirubin (TBil), blood urea nitrogen (BUN) and Scr were measured. The estimated glomerular filtration rate (eGFR) was calculated within 1 week before the MRI. Patients with liver cirrhosis were divided into two subgroups: cirrhosis with normal renal function (NR; the cirrhosis-NR group) and cirrhosis with RD (the cirrhosis-RD group). Cirrhosis-RD was diagnosed according to the criteria for hepatorenal syndrome-AKI (4).

Exclusion criteria were primary kidney diseases such as nephrotic syndrome and glomerular nephritis; hypertension, diabetic mellitus, or gout disease; serious systemic or organic lesions; contraindications for MRI; and use of medications such as diuretics, angiotensin-converting enzyme inhibitors, or angiotensin receptor blockers before MRI examination. Four patients with incomplete images and two with image artifacts were also excluded.

Twenty-three age and body mass index (BMI) matched healthy volunteers with no history of renal disease, diabetes, hypertension, or vascular disease were recruited as the normal control (NC) group. All NCs had normal BUN, Scr and eGFR levels and no renal lesions according to conventional T2-weighted MRI. **Table 1** lists the demographic data for all participants.

**Table 1:** Demographic and clinical characteristics of the patients and controls.

	NC group	Cirrhosis-NR group	Cirrhosis-RD group	F value	*p* value
Number	23	27	10		
Age	44.09 ± 13.91	49.78 ± 11.38	49.90 ± 11.63	1.498	0.232
Sex (male/female)	10/13	7/20	2/8	2.270	0.132
BMI	23.39 ± 3.04	22.89 ± 2.91	23.20 ± 2.74	0.185	0.832
BUN (mmol/L)	4.42 ± 1.19	4.80 ± 1.61	9.25 ± 5.72	12.805	0.002^*^
Scr (µmol/L)	67.30 ± 12.70	61.74 ± 14.43	154.80 ± 66.59	40.021	<0.001^*^
eGFR (mL/min/1.732 m^2^)	104.67 ± 31.21	116.15 ± 28.54	47.72 ± 19.95	21.513	<0.001^*^
ALT (U/L)	21.74 ± 10.28	27.32 ± 16.97	35.98 ± 29.75	1.845	0.398
AST (U/L)	19.39 ± 5.21	46.88 ± 29.76	43.34 ± 19.29	29.094	<0.001^*^
TBil (mmol/L)	13.98 ± 3.38	88.59 ± 88.32	137.47 ± 182.55	23.601	<0.001^*^

* p values < 0.05 indicate statistical significance.

NC, normal control; Cirrhosis-NR, cirrhosis with normal renal function; Cirrhosis-RD, cirrhosis with renal dysfunction; BMI, body mass index; BUN, blood urea nitrogen; Scr, serum creatine; eGFR, estimated glomerular filtration rate; ALT, alanine transaminase; AST, aspartate aminotransferase; TBil, total bilirubin.

Data are means ± standard deviation.

Except for sex, which was compared using χ2 tests, clinical characteristics among the groups were compared using one-way analysis of variance.

### MRI technique

All participants were required to fast for ≤6 h before MRI. All MRI examinations were performed on a 3T MR system (MAGNETOM Prisma, Siemens Healthcare, Erlangen, Germany) equipped with an eighteen-channel body array coil. Conventional T2-weighted imaging-interpolated breath-hold examinations were performed to evaluate renal lesions. T1 mapping and ASL were used to quantify renal microstructure and perfusion changes. **Table 2** lists the scanning parameters of all MRI sequences.

**Table 2:** MRI protocol parameters.

	T2-weighted imaging	T1 mapping	ASL
Orientation	Axial	Coronal	Coronal
Respiratory pattern	Respiratory trigger	Breath-hold	Free-breathing
TR/TE (ms)	2200/113	3/1.32	6000/20.84
Field of view (mm^2^)	400×262	380×380	300×150
Slice thickness (mm)	6	4	6
Matrix	448×206	128×102	64×32
Voxel size (mm^3^)	0.9×0.9×6	1.5×1.5×4	4.7×4.7×6
Acceleration factor	3	2	1
Acquisition time	2 min 20 sec	20 sec	4 min 6 sec

ASL, arterial spin labeling.

T1 mapping was performed using a 2D Look-Locker sequence. The sequence performed using a prototype inversion recovery snapshot FLASH acquisitions after a 180° inversion pulse, providing images at multiple inversion times (16 TIs at 104, 293, 482, 671, 860, 1049, 1238, 1427, 1616, 1805, 1994, 2183, 2372, 2561, 2750, and 2939 ms). Four slices centered on the renal hilum were obtained for each patient. The T1 parameter map was generated inline after data acquisition by the scanner, and was based on a compartment model processing images pixel-by-pixel.

ASL was obtained using a prototypical three-dimensional TGSE research sequence with a pseudo-continuous ASL (pCASL) scheme. Abdominal aorta was labeled with a labeling duration of 1500 ms and post-labeling delay of 3000 ms. One segment with 20 repetitions was performed. Perfusion map was estimated on a pixel basis using the following formula:


$$\begin{array}{c} \text{Δ}M\left(t\right)=2\frac{M_{0}}{\lambda }fT_{1}^{'}\alpha \text{exp}(\frac{-\text{Δ}t}{T_{1,\text{blood}}})\cdot \text{ exp}(\frac{-(t-\tau -\text{Δ}t}{T_{1}^{'}})\cdot (1-\text{exp}(\frac{-\tau }{T_{1}^{'}})),\\ \text{ with }\frac{1}{T_{1}^{'}}=\frac{1}{T_{1}}+\frac{f}{\lambda }, \end{array}$$

where *f* is the perfusion rate (in mL/100g/minute); λ represents the blood-tissue partition coefficient (0.9 mL/100 g); *M*_0_ is the equilibrium magnetization intensity; α is the inversion efficiency (0.98); Δ *t* is the arrival time of labeled blood (assumed to be 750 ms); T_1,blood_ is the longitudinal relaxation time of arterial blood; τ is the labeling time. Images were acquired in free breathing mode, and motion correction performed with motion compensation using 3D rigid and elastic registration algorithms.

### Imaging analysis

Two experienced radiologists (with 11 and 3 years of experience each) who were blinded to the clinical data evaluated all MRI images. Renal injury in patients with liver cirrhosis is a systemic disease, main reason is the hemodynamic changes, so we assume the injury was similar between the two kidneys. MRI measurements were taken on bilateral kidneys separately. Three sections nearest the renal hilum were selected to draw the region of interest (ROI). For each selected section, one free-hand ROI (5–9 cm^2^) covering the entire renal cortex was manually drawn on both the T1 and RBF images, and three ellipsoid ROIs (0.2–0.4 cm^2^) were manually and separately placed on the upper, middle, and lower poles of the renal medulla on the T1 image.

### Statistical analysis

Statistical analyses were performed using SPSS (version 25.0; IBM), MedCalc (version 19.0.4) and Python (version 3.9) software. Normally distributed variables are presented as means ± standard deviation and non-normally distributed variables are presented as medians (median, absolute deviation) after performing the Shapiro-Wilk test. The male/female ratio for each group was calculated via the chi-square test. Age, BMI, biochemical indices and renal MRI parameters for each group were compared using one-way analysis of variance (normal distribution and equal variances) or the Kruskal-Wallis test (non-normal distribution or equal variances). *Post hoc* multiple pairwise comparisons were performed using Bonferroni tests. Pearson/Spearman correlation tests were used to assess correlations between renal MRI parameters (cT1, mT1, and RBF) and biochemical indices (BUN, Scr, eGFR, ALT, AST and TBil). Receiver operating characteristic (ROC) curves were used to evaluate the diagnostic performances of the MRI parameters. Areas under the curve (AUCs) were compared using the DeLong method. A classification and regression tree (CART) analysis using cross-validation was performed using renal MRI parameters to identify renal impairment in patients with cirrhosis. CART analysis results are presented as a decision tree. Inter-reader reliability for measuring MRI imaging data was calculated using intraclass correlation coefficients (ICCs) and Bland-Altman analysis. *p*<0.05 was considered statistically significant.

## Results

### Clinical characteristics

Twenty-three healthy volunteers (NCs), 27 patients with cirrhosis and NR, and 10 patients with cirrhosis and RD were included in this study (**Table 1**). Sex, age, BMI and ALT did not significantly differ among the three groups (all *p*>0.05). BUN, Scr, and eGFR did not significantly differ between the NCs and cirrhosis-NR group (all *p*>0.05) but were significantly higher in the cirrhosis-RD group than in the cirrhosis-NR group (all *p*<0.05). AST and TBil did not differ between the cirrhosis subgroups (all *p*>0.05) but were significantly higher in the subgroups than in the NCs (all *p*<0.01).

### T1 mapping and ASL parameters

**Figure 1:**
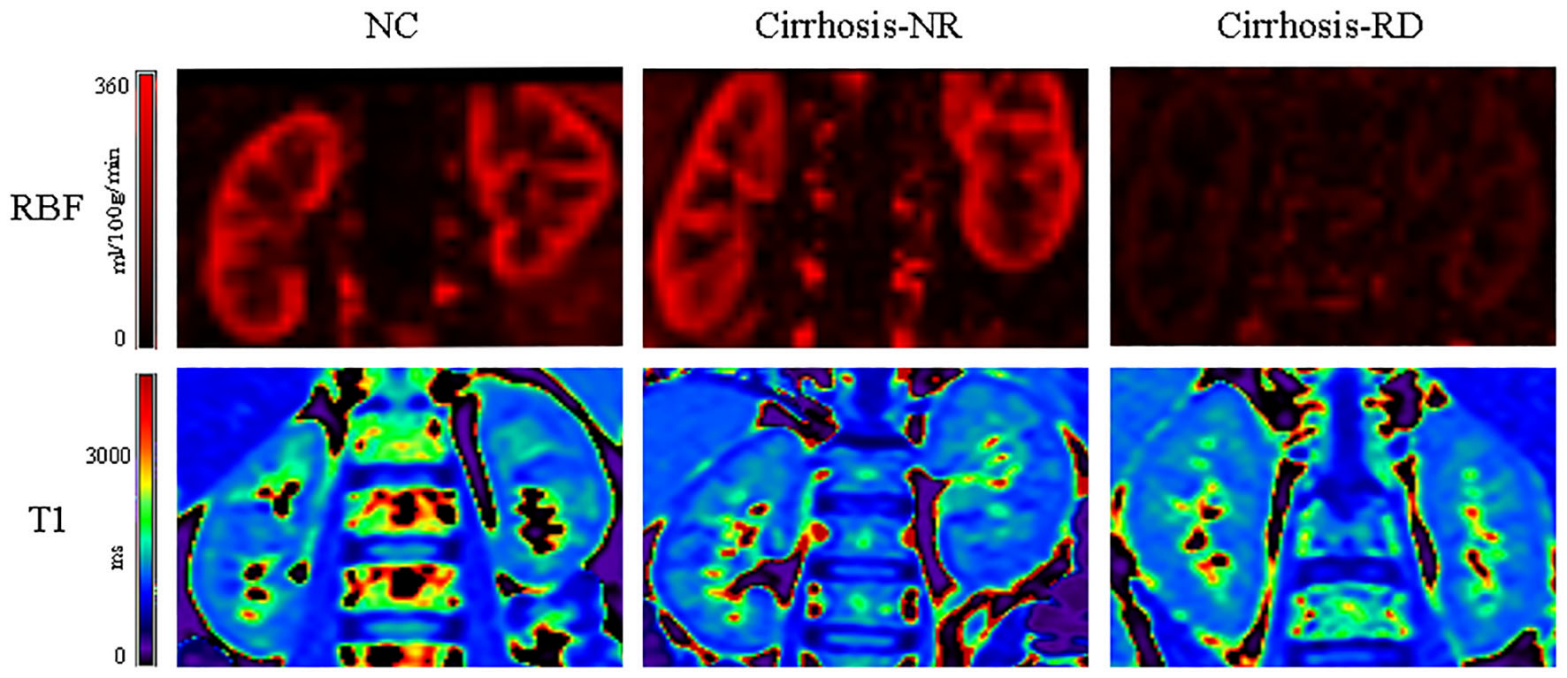
Representative examples of ASL and T1 mapping parameters in NCs and patients. *NC,* normal control; Cirrhosis-NR, cirrhosis with normal renal function; Cirrhosis-RD, cirrhosis with renal dysfunction.

**Figure 1** shows representative T1 and RBF maps of the two subgroups and the NCs. **Table 3** presents the MRI parameters of each group. The renal cortical T1 (cT1) values, medullary T1 (mT1) values and cortical RBF values showed no significant differences between the left and right kidneys (all P > 0.05; **Supplementary Table 1**). The average measurements of both sides of each participant’s kidney were used for the final analysis.

**Table 3:** Renal T1 and RBF values of the study groups^*^.

Group	cT1 (ms)	mT1 (ms)	RBF (mL/100 g/min)
NCs	1195.50 (14.33)	1523.13 (51.54)	312.93 (56.36)
Cirrhosis-NR	1138.17 (34.67)	1450.91 (57.09)	324.27 (61.50)
Cirrhosis-RD	1168.00 (21.42)	1455.45 (51.19)	152.88 (61.80)
*p* value	<0.001^*^	<0.001^*^	<0.001^*^

^*^cT1 values are shown as medians (absolute deviation). mT1 and RBF values are shown as mean (standard deviation). NC, normal control; Cirrhosis-NR, cirrhosis with normal renal function; Cirrhosis-RD, cirrhosis with renal dysfunction; cT1, cortical T1 values; mT1, medullary T1 values; RBF, renal blood flow.

**Figure 2:**
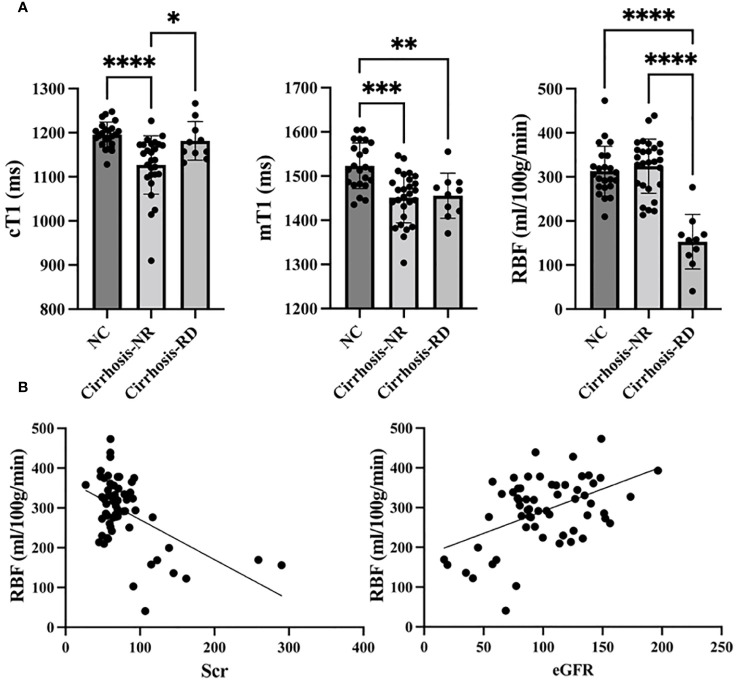
Comparison of T1 mapping and ASL parameters between controls and patients **(A)**. Correlations between RBF and Scr and eGFR **(B)**. *NC,* normal control; *Cirrhosis-NR,* cirrhosis with normal renal function; *Cirrhosis-RD,* cirrhosis with renal dysfunction; *Scr,* serum creatinine; *eGFR,* estimated glomerular filtration rate. ^*^ *p*<0.05, ^**^ *p*<0.01, ^***^ *p*<0.001, ^****^ *p*<0.0001.

The renal cortical T1 (cT1) values, medullary T1 (mT1) values and cortical RBF differed significantly among the three groups (all *p*<0.001). Subsequent *post hoc* tests confirmed significant differences between the NCs and cirrhosis-NR group for renal cT1 and mT1 values (all *p*<0.001), between the NCs and cirrhosis-RD group for renal mT1 values (*p*<0.01), and between both cirrhosis subgroups for renal cT1 and RBF values (all *p*<0.05; **Figure 2A**). Cortical RBF values were weakly to moderately correlated with Scr (r= −0.334, *p*=0.009) and eGFR (r=0.483, *p*=0.003; **Figure 2B**).

### Diagnostic performances of the T1 and RBF

**Figure 3:**
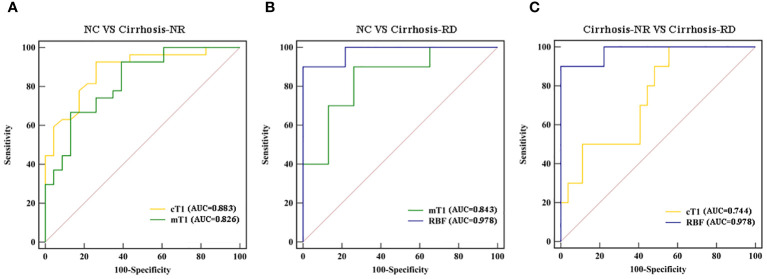
Receiver operating characteristic curves for discriminating **(A)** cirrhosis-NR patients from NCs, **(B)** cirrhosis-RD patients from NCs, and **(C)** cirrhosis-RD patients from cirrhosis-NR patients **(C)**. *NC,* normal control; *Cirrhosis-NR,* cirrhosis with normal renal function; *Cirrhosis-RD,* cirrhosis with renal dysfunction.

**Table 4** and **Figure 3** show the ROC curves of the T1 and RBF values for predicting renal impairment in patients with liver cirrhosis. Both renal cT1 and mT1 had high diagnostic performances in differentiating the cirrhosis-NR group and NCs (AUCs: 0.883 and 0.826, respectively; *p*=0.3362). Renal RBF performed better than did mT1 in differentiating between the cirrhosis-RD group and NCs (AUCs: 0.978 and 0.843, respectively; *p*=0.0076) and better than did cT1 in differentiating between the cirrhosis-RD and cirrhosis-NR groups (AUCs: 0.978 and 0.744, respectively; *p*=0.0187).

**Table 4:** Diagnostic characteristics of the renal T1 and ASL parameters.

Parameters	AUC (95% CI)	Cut-off value	Sensitivity (%)	Specificity (%)
NC (n=23) vs cirrhosis-NR (n=27)				
cT1	0.883 (0.761–0.957)	≤1183.8	92.6%	73.9%
mT1	0.826 (0.693–0.919)	≤1475.7	66.7%	87.0%
NC (n=23) vs cirrhosis-RD (n=10)				
mT1	0.843 (0.675–0.946)	≤1485.7	90%	73.9%
RBF	0.978 (0.856–1.000)	≤199.3	90%	100.0%
Cirrhosis-NR (n=27) vs cirrhosis-RD (n=10)				
cT1	0.744 (0.575–0.873)	≤1126.8	100%	44.4%
RBF	0.978 (0.866–1.000)	≤199.3	90%	100%

AUC, area under the curve; CI, confidence interval; NC, normal control; Cirrhosis-NR, cirrhosis with normal renal function; Cirrhosis-RD, cirrhosis with renal dysfunction; cT1, cortical T1 values; mT1, medullary T1 values; RBF, renal blood flow.

Cut-off values for cT1 and mT1 are in ms; RBF is mL/100 g/min.

### CART analysis

**Figure 4:**
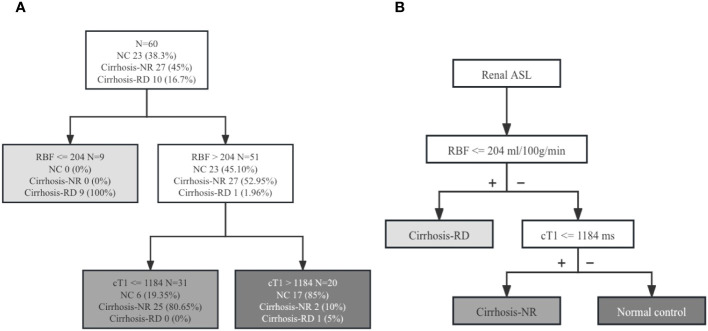
**(A)** Diagnostic algorithm of 60 patients. Classification rule for renal RBF and cT1 values based on the intuitive tree generated by CART analysis. **(B)** New diagnostic algorithm for evaluating renal injury in patients with cirrhosis. *ASL,* arterial spin labeling; *NC,* normal control; *Cirrhosis-NR,* cirrhosis with normal renal function; *Cirrhosis-RD,* cirrhosis with renal dysfunction; *cT1,* cortical T1 values; *RBF,* renal blood flow.

The classification tree obtained via multivariate CART analysis included renal T1 and RBF values and suggested the best sequence of their application in a diagnostic algorithm. After including renal cT1, mT1, and cortical RBF, only renal cT1 and RBF were selected to create the classification tree (**Figure 4**). In the first step, the cortical RBF decrease (≤ 204 mL/100 g/min) was used to classify nine patients with cirrhosis (90%) as potentially having RD. For the remaining 51 participants who had no decrease in RBF (> 204 mL/100 g/min), renal cT1 was evaluated in the second step. Twenty-five participants exhibited decreased renal cT1 values (cortical T1 ≤ 1184 ms), indicating cirrhosis-NR, and 17 (73.9%) exhibited high renal cT1 (> 1184 ms), indicating they were the NCs. Thus, the diagnostic algorithm correctly identified cirrhosis-RD in 9 of 10 patients and cirrhosis-NR in 25 of 27 patients, yielding 85% accuracy. **Table 5** shows the diagnostic performances for detecting NCs, cirrhosis-NR and cirrhosis-RD. Renal mT1 and cortical RBF were manually selected to create the classification tree with 80% accuracy (**Supplementary Figure 1**).

**Table 5:** Diagnostic characteristics of the classification tree based on T1 and ASL parameters.

Class	AUC	Sensitivity (%)	Specificity (%)	Accuracy (%)
NC	0.861	73.91%	91.89%	85%
Cirrhosis-NR	0.882	92.59%	81.82%	86.67%
Cirrhosis-RD	0.981	90%	100%	98.33%

AUC, area under the curve; NC, normal control; Cirrhosis-NR, cirrhosis with normal renal function; Cirrhosis-RD, cirrhosis with renal dysfunction.

### Interobserver variability of the MRI measurements

The ICCs of the cT1, mT1 and cortical RBF values were 0.936 (95% confidence interval [CI], 0.894–0.962), 0.888 (95% CI, 0.812–0.933), and 0.947 (95% CI, 0.912–0.968), respectively. **Supplementary Figure 2** shows the Bland-Altman plots.

## Discussion

We evaluated the value of T1 mapping and ASL parameters in detecting renal injury and identifying RD in patients with liver cirrhosis. Patients with cirrhosis-NR had decreased renal parenchymal T1 values compared with those of the NCs, thus indicating the value of T1 mapping in detecting renal impairment before RD. Furthermore, the RBF values derived from ASL reflected RD in patients with cirrhosis with high diagnostic efficiency. The diagnostic algorithm based on cortical RBF and T1 values allowed detecting renal injury in patients with cirrhosis.

Comparing the cortical RBF among the three groups showed that the RBF was significantly lower in patients with cirrhosis-RD than in the other two groups, but similar between the NCs and patients with cirrhosis-NR. Thus, changes in renal perfusion may not be obvious in patients with cirrhosis-NR, but when Scr levels indicate abnormal renal function, renal perfusion is consistently significantly decreased. This is consistent with the mechanism of renal injury potentially involved in hepatorenal syndrome-AKI in patients with cirrhosis. Cirrhosis leads to portal hypertension and systemic vasodilation followed by reduced EABV, after which, systemic vasoconstrictor pathways are activated to increase the EABV, resulting in renal vasoconstriction and consequently reduced RBF (9, 20). In earlier stages, renal perfusion can be maintained. Disruption of this balance leads to renal dysfunction. Our previous study with rats showed that ASL can be a noninvasive tool for assessing renal injury induced by cholestatic and drug toxic cirrhosis (13, 18).

In addition to circulatory dysfunction, systemic inflammation, adrenal insufficiency and bile acids play important roles in renal injury development and can result in direct tubular damage. The pathology of kidney injury involves tubule dilation, brush border destruction, vacuole degeneration, interstitial edema, inflammatory cell infiltration, tube casting, nuclear pyknosis and fibrosis. Cardiac T1 mapping images have been used in clinical practice to quantify myocardial edema and fibrosis (21, 22). Previous studies of renal T1 mapping evaluated ischemia-induced AKI following solid organ transplantation and renal fibrosis resulting from chronic renal disease and unilateral ureteral obstruction (15, 16, 23, 24). These studies showed increased renal T1 values after renal injury, which was associated with cellular swelling and interstitial edema during the acute phase and water accumulation in enlarged interstitial spaces during renal fibrosis.

We used T1 mapping to reveal renal impairment in patients with liver cirrhosis. Both renal cT1 and mT1 values were significantly reduced in patients with cirrhosis-NR compared with those in NCs. Factors that contribute to reduced T1 values include lipid deposition (25), iron deposition (26) and high glycogen concentrations (27). Disturbances in iron regulation have been described in chronic liver diseases (28, 29). Iron deposition in renal tissue may contribute to the progression of chronic renal disease (30). Thus, lower renal T1 values may be due to iron accumulation. Further studies are needed to investigate the pathological changes in the kidneys during cirrhosis.

Renal cT1 values were significantly higher in patients with cirrhosis-RD than in those with cirrhosis-NR and similar to those of NCs, possibly because renal injury in this stage prolongs T1, and the effects of the prolonging and shortening were offset. Thus, renal cT1 values did not differ between patients with cirrhosis-RD and NCs. However, Özkök et al. (19) found that both renal cT1 and mT1 values were significantly higher in patients with chronic hepatitis B viral infection than in NCs. In that study, renal mT1 values were significantly shorter than cT1 values in both patients and NCs, which is inconsistent with the normal physiological structure of the kidney. Another study found that the water content was significantly higher in the renal medulla than in the cortex, resulting in longer T1 time in the medulla than in the cortex in NC (24). These results require further confirmation.

Our diagnostic analysis showed that cT1 and mT1 values had good diagnostic performances for detecting renal changes between NCs and patients with cirrhosis-NR, suggesting that T1 is a valuable MRI biomarker for detecting early renal impairment during cirrhosis. Renal RBF had good diagnostic performance for detecting renal changes between NCs and patients with cirrhosis-RD and between patients with cirrhosis-NR and cirrhosis-RD, suggesting that RBF is also a valuable MRI biomarker for detecting RD during cirrhosis. Renal RBF was mildly to moderately correlated with Scr and eGFR. Our diagnostic algorithm based on RBF and cT1 showed good diagnostic performance with 85% accuracy. Therefore, ASL combined with T1 mapping can be used as a noninvasive tool to monitor renal abnormalities in patients with liver cirrhosis.

This study had some limitations. First, the sample size was relatively small, particularly for patients with cirrhosis-RD. Second, RBF derived from ASL is not the gold standard and lacks comparison with more accurate methods. Third, direct histological evidence of renal T1 and RBF changes was lacking. Future longitudinal studies should track these changes over time.

In conclusion, renal T1 values were decreased in patients with cirrhosis-NR, whereas RBF values were decreased only in patients with cirrhosis-RD. T1 mapping and ASL MRI can be used as noninvasive tools to reveal early renal changes and RD during cirrhosis. Our new diagnostic algorithm based on cT1 and RBF may potentially detect renal impairment in patients with cirrhosis.

## Data Availability

The original contributions presented in the study are included in the article/**Supplementary Material**. Further inquiries can be directed to the corresponding author.
