# Supplementary material for: T1 mapping combined with arterial spin labeling MRI to identify renal injury in patients with liver cirrhosis
Source: Front Endocrinol (Lausanne). 2024 Aug 9;15:1363797. doi: 10.3389/fendo.2024.1363797 (PMC11341387; doi:10.3389/fendo.2024.1363797)
Supplement: Supplementary file 1 [file DataSheet_1.docx]

**Appendix**

**
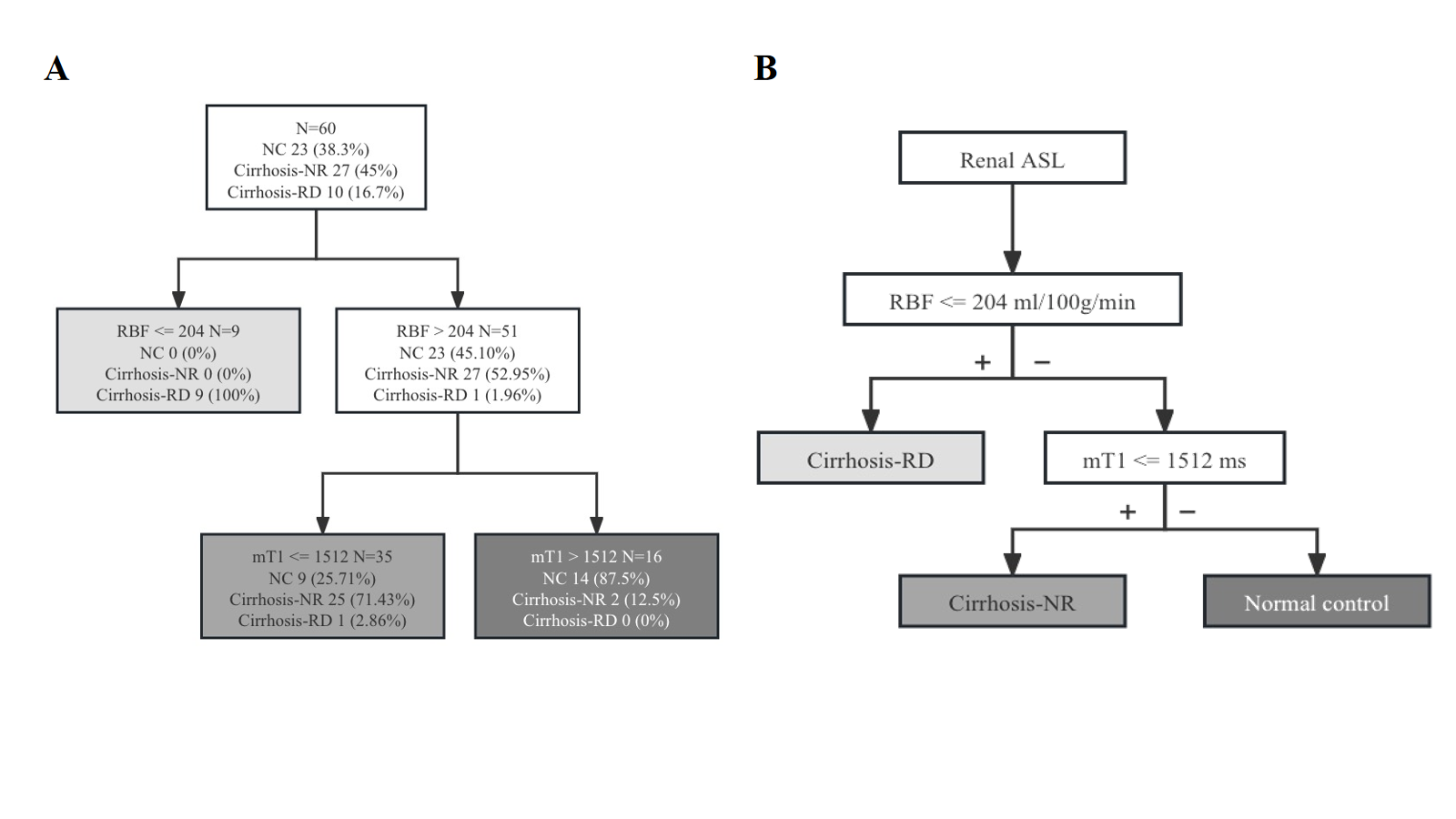
**

**Figure 1:** (A) Diagnostic algorithm of 60 patients. Classification rule for renal RBF and mT1 values based on the intuitive tree generated via CART analysis. (B) New diagnostic algorithm for evaluating renal injury performed on patients with cirrhosis. *ASL:* arterial spin labeling, *NC:* normal control, *Cirrhosis-NR:* cirrhosis with normal renal function, *Cirrhosis-RD:* cirrhosis with renal dysfunction, *mT1:* medullaryT1 values, *RBF:* renal blood flow.

**
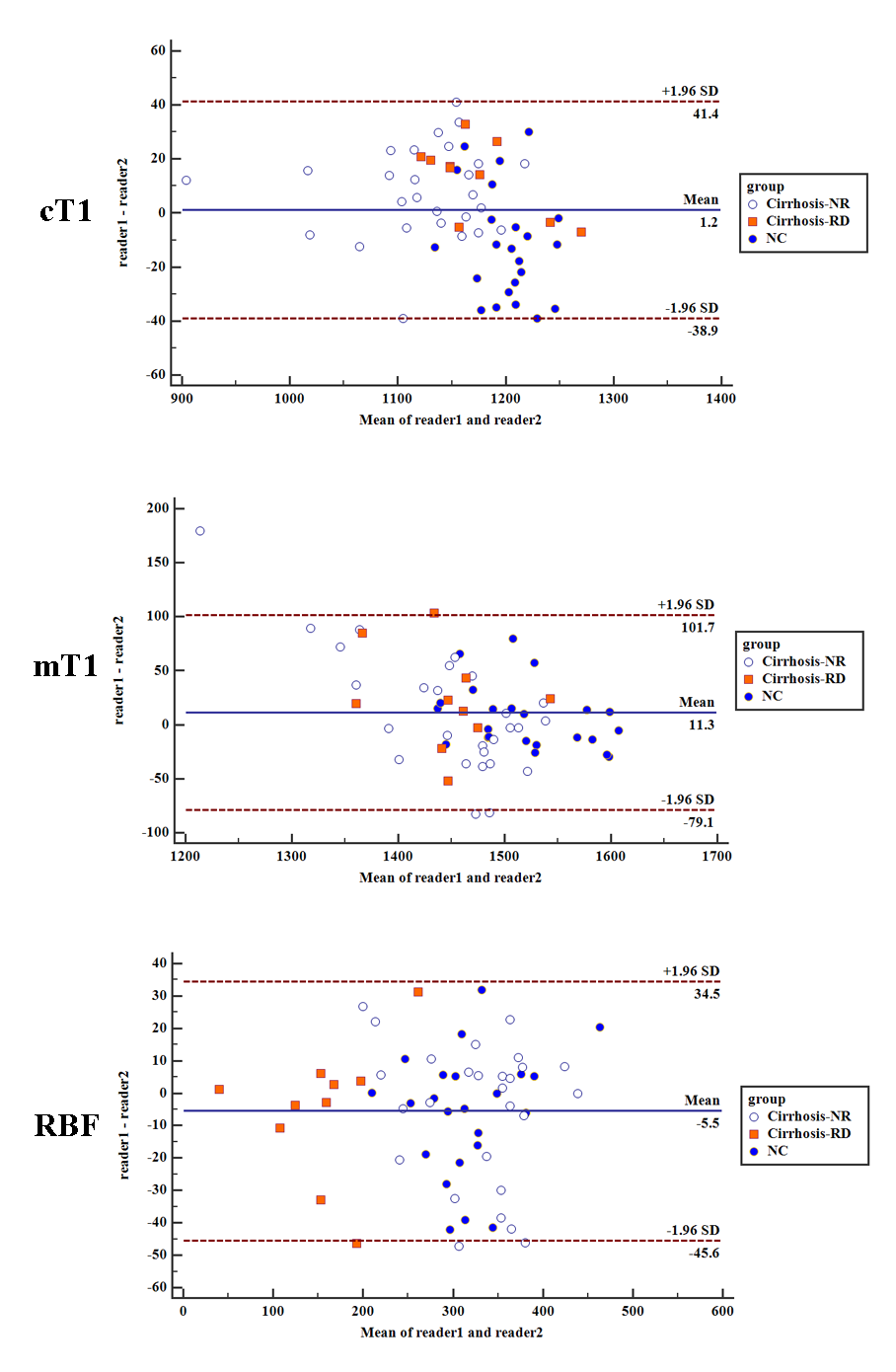
**

**Figure 2:** Bland-Altman plots for two readers. The solid blue line represents the mean of the difference. Dotted red lines represent 95% confidence intervals.

**Table 1.** Renal T1 and RBF values of the right and left kidney

| Group | cT1 (ms) | mT1 (ms) | RBF (mL/100 g/min) |
| --- | --- | --- | --- |
| Kidney-left | 1165.60 ± 65.61 | 1491.30 ± 76.78 | 291.10 ± 85.19 |
| Kidney-right | 1158.91 ± 93.21 | 1467.41 ± 96.75 | 291.62 ± 86.86 |
| t | 0.481 | 1.545 | -0.315 |
| *p* value | 0.632 | 0.128 | 0.745 |

^*^*cT1:* cortical T1 values, *mT1:* medullary T1 values, *RBF:* renal blood flow
